# Comparing the Limbic–Frontal Connectome across the Primate Order: Conservation of Connections and Implications for Translational Neuroscience

**DOI:** 10.1523/JNEUROSCI.0377-25.2026

**Published:** 2026-01-30

**Authors:** Davide Folloni, Lea Roumazeilles, Katherine L. Bryant, Paul R. Manger, Mads F. Bertelsen, Alexandre A. Khrapitchev, Peter H. Rudebeck, Rogier B. Mars

**Affiliations:** ^1^Nash Family Department of Neuroscience and Friedman Brain Institute, Icahn School of Medicine at Mount Sinai, New York, New York 10029; ^2^Lipschultz Center for Cognitive Neuroscience, Icahn School of Medicine at Mount Sinai, New York, New York 10029; ^3^Vita-Salute San Raffaele University, Milan 20132, Italy; ^4^Wellcome Centre for Integrative Neuroimaging Nuffield Department of Clinical Neurosciences, John Radcliffe Hospital, University of Oxford, Oxford OX3 9DU, United Kingdom; ^5^Laboratoire de Psychologie Cognitive, Aix-Marseille Université, 13331 Marseille Cedex 3, France; ^6^School of Anatomical Sciences, Faculty of Health Sciences, University of Witwatersrand, Johannesburg 2193, South Africa; ^7^Centre for Zoo and Wild Animal Health, Copenhagen Zoo, Frederiksberg 2000, Denmark; ^8^Department of Oncology, Cancer Research UK and Medical Research Council Oxford Institute for Radiation Oncology, University of Oxford, Oxford OX3 7DQ, United Kingdom; ^9^Donders Institute for Brain, Cognition and Behaviour, Radboud University, Nijmegen 6500 HD, the Netherlands

**Keywords:** amygdalofugal, comparative, connectivity, frontal cortex, limbic system, translational neuroscience, uncinate

## Abstract

The interaction of the limbic system and frontal cortex of the primate brain is important in many affective behaviors. For this reason, it is heavily implicated in a number of psychiatric conditions. This system is often studied in the macaque monkey, the most largely used nonhuman primate model species. However, how evolutionary conserved this system is and how well results obtained in any model species translate to the human brain can only be understood by studying its organization across the primate order. Here, we present an investigation of the topology of limbic–frontal connections across seven species, representing all major branches of the primate family tree: humans (13 females, 11 males), chimpanzee (1 female), gorilla (1 male), gibbon (1 male), macaque (1 female, 2 males), squirrel monkey (1 female, 2 males), and lemur (3 males). We show that dichotomous organization of amygdalofugal and uncinate connections with the frontal cortex is conserved across all species. Subgenual connectivity of the cingulum bundle, however, seems less prominent in prosimian and New World monkey brains. These results inform both translational neuroscience and primate brain evolution.

## Significance Statement

The interaction between the limbic system and the frontal cortex is critical for affective behaviors and is implicated in psychiatric conditions. While often studied in macaques, understanding how conserved these circuits are across primates is essential for translational relevance. Here, we investigate limbic–frontal connections across seven primate species, spanning all major evolutionary branches. We demonstrate that the dichotomous organization of amygdalofugal and uncinate pathways is conserved, while subcallosal cingulate connectivity of the cingulum bundle is less prominent in prosimian and New World monkeys. These findings provide key insights into the evolution of primate brains and enhance our understanding of the translational potential of nonhuman primate models for studying human brain function and disorders.

## Introduction

The limbic system and frontal cortex form an interconnected system of brain areas that are affected in disorders of mood, motivation, and anxiety (Mayberg, 2001; Kenwood et al., 2022; Klein-Flügge et al., 2022). Reciprocal connectivity between the parts that form this system are essential to support their roles in associative learning, decision-making, and behavioral control (Murray and Wise, 2010; Saez et al., 2015; Munuera et al., 2018). As such, these connections are often the target of clinical interventions such as deep-brain stimulation (Mayberg et al., 2005; Johansen-Berg et al., 2008; Riva-Posse et al., 2014). Advances in noninvasive stimulation of deep cortical structures (Fouragnan et al., 2019; Folloni et al., 2019b; Verhagen et al., 2019; Folloni, 2022) mean that functional interactions between connectional systems can potentially be targeted without surgical interventions. However, to advantageously and judiciously use these advances, a better understanding of the limbic–frontal connectome is required.

Much of our knowledge of these systems is based on invasive work in nonhuman animals, using techniques that cannot be ethically and legally applied to humans. The development of neuroimaging techniques that can be applied to both humans and nonhuman animals with increasing levels of resolution has enabled formal tests of how well results obtained in model species translate to humans (Mars et al., 2014). For example, comparative diffusion MRI-based tractography work has concentrated on assessing the principles of frontal cortex organization and connectivity in humans and macaque monkeys (Croxson et al., 2005; Sallet et al., 2013; Barrett et al., 2020). Recent investigations demonstrate how such methods can also be used to identify even very specific limbic–frontal connections. For instance, the amygdalofugal pathway (AmF), a small pathway running between the amygdala and prefrontal cortex (PFC; Nauta, 1961, 1962; Aggleton et al., 1980; Lehman et al., 2011), could be detected in humans using tractography, as long as the approach is guided by anatomical knowledge obtained in macaques using both invasive tracers and tractography (Folloni et al., 2019a). Comparative neuroimaging can thus be a valuable tool for investigating how limbic–frontal circuits vary across species.

Neuroimaging has the additional advantage that it can be applied to a larger number of species than can often be used in comparative neuroanatomical studies. Comparisons do not have to be limited to studies of humans and macaques and can be extended to a broader range of primate species, allowing the characterization of patterns of evolutionary consistency or change. Understanding such patterns will lead to a better understanding of how well results obtained in any model species are likely to generalize to the species of interest, which in most cases is the human. The increased availability of MRI datasets from nonhuman primates (Milham et al., 2018; Tendler et al., 2022), combined with new analytical frameworks (Mars et al., 2021), makes such studies feasible.

**Figure 1.**
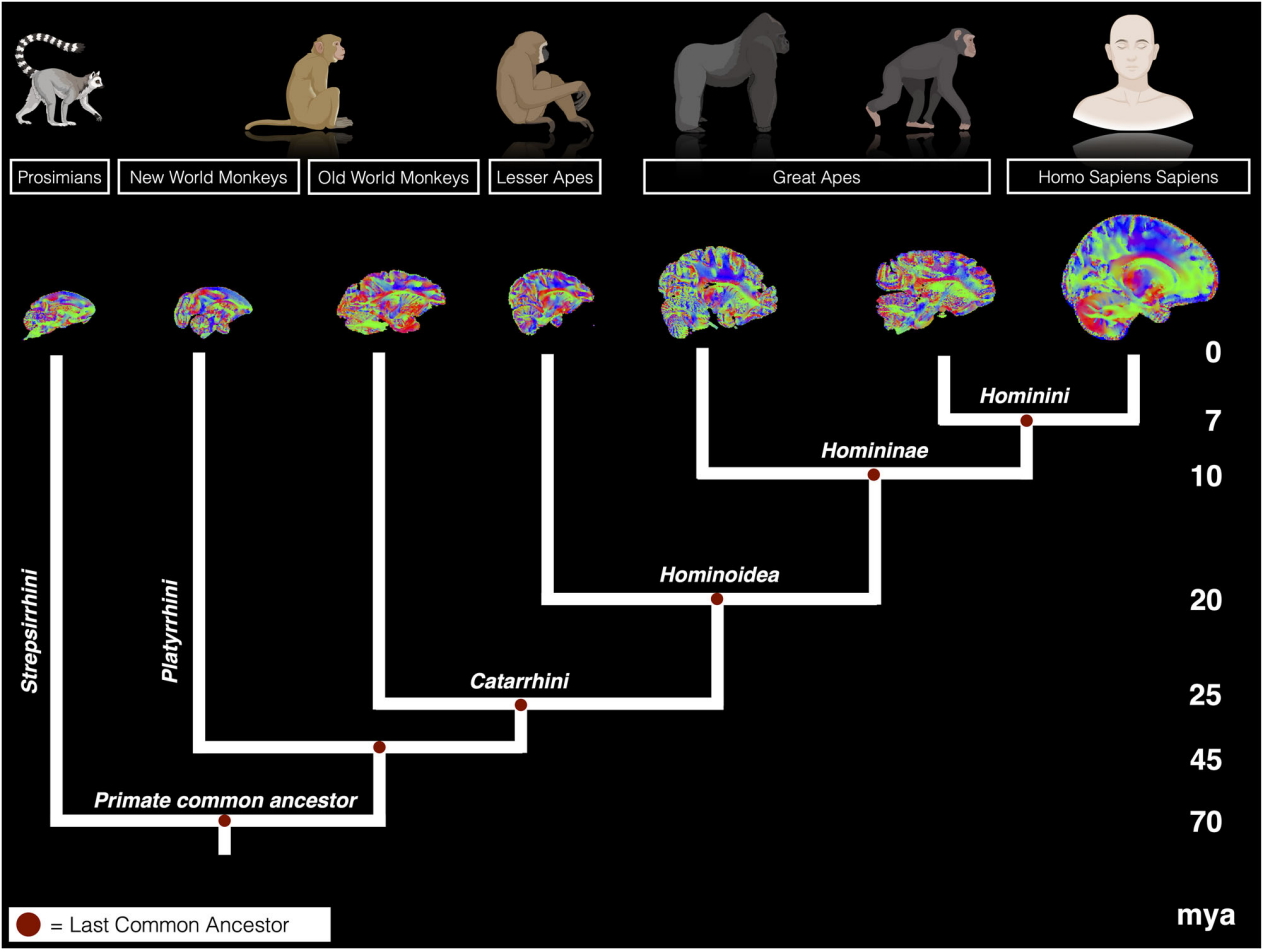
Primate family tree. Phylogram showing evolutionary relationships of prosimians, Old and New World monkeys, lesser and great apes, and humans (Ni et al., 2019; MacLatchy et al., 2020; Eldridge et al., 2021; Preuss and Wise, 2022). Brains are not to scale. Mya, million years ago.

The frontal cortex is thought to be quite variable across primate species, both in term of its relative size and connectivity with other brain regions (Passingham and Smaers, 2014; Donahue et al., 2018; Mars et al., 2018). Therefore, we set out to compare the major pathways that connect to the frontal cortex from the limbic system. It is possible that organizational variances of these frontal cortical areas among primates may be associated with differently organized patterns of connectivity. Alternatively, the course and organization of the amygdala–cingulate–prefrontal circuits may be conserved across primate species. To test these possibilities, we reconstructed the cingulum bundle (CB), ventral AmF, and the uncinate fasciculus (UF)across an unprecedented range of primate species using probabilistic tractography (Fig. 1). We observed that, at the level of diffusion-tractography estimates of anatomical connectivity, the amygdala–cingulate–prefrontal circuits are similar in their course and overall pattern of organization not only in anthropoid brains but also in prosimians.

## Materials and Methods

### Data acquisition and preprocessing

#### Human data

Human in vivo diffusion MRI data were provided by the Human Connectome Project (HCP), WU-Minn Consortium (Principal Investigators, David Van Essen and Kamil Ugurbil; 1U54MH091657) funded by the 16 NIH Institutes and Centers that support the NIH Blueprint for Neuroscience Research and by the McDonnell Center for Systems Neuroscience at Washington University (Van Essen et al., 2013). The minimally preprocessed datasets of the first 24 subjects from the Q2 public data release were used (age range 22–35 years; 13 females). Data acquisition and preprocessing methods are detailed in Uğurbil et al. (2013), Sotiropoulos et al. (2013), and Glasser et al. (2013). The diffusion data were collected at a 1.25 mm isotropic resolution across the entire brain on a customized 3 T Siemens Skyra scanner using a monopolar Stejskal-Tanner diffusion scheme with a slice-accelerated EPI readout. Sampling in *q*-space included three shells at *b* = 1,000, 2,000, and 3,000 s/mm^2^. For each shell 90 diffusion gradient directions and six nondiffusion-weighted images (*b* = 0 s/mm^2^) were acquired with reversed phase-encoding direction for TOPUP distortion correction (Andersson et al., 2003). Voxel-wise model fitting of diffusion orientations was performed using FSL's bedpostX to fit a crossing fiber model to the data (Behrens et al., 2007). A multishell extension was used to reduce overfitting of crossing fibers due to nonmonoexponential diffusion decay (Jbabdi et al., 2012). Up to three fiber orientations per voxel were allowed.

#### Chimpanzee and gorilla

Whole-brain samples from one chimpanzee (*Pan troglodytes*, one female, 54 years of age at the time of death) and one gorilla (*Gorilla gorilla*, male, 12 years of age at the time of death) were obtained from the Primate Brain Bank and the London Zoological Society. The samples were acquired from animals that died for reasons unrelated to this research project. Details of sample preparation, scanning procedure, and data preprocessing have been communicated before (Roumazeilles et al., 2020; Bryant et al., 2021). In brief, samples were stored in formalin, rehydrated using a phosphate-buffered saline (PBS) solution 1 week prior to scanning and placed in fluorinert for the scanning procedure. Samples were imaged using a 7 T whole-body scanner with a 28-channel knee coil (QED). Diffusion MRI data were acquired using a 3D diffusion-weighted steady–state free precession (DW-SSFP) pulse sequence (McNab et al., 2009). DW-SSFP data comprising of 240 diffusion-weighted (*q* = 300 cm^−1^; resolution = 0.6 mm isotropic) and 6 nondiffusion-weighted (*q* = 20 cm^−1^) imaging volumes were acquired over the whole postmortem brains. To account for the T1, T2, and flip-angle dependencies of the DW-SSFP signal (Buxton, 1993; McNab and Miller, 2010), T1, T2, and B1 datasets were acquired via a turbo inversion-recovery (TIR), turbo spin-echo (TSE), and actual flip-angle imaging (AFI) acquisition (Yarnykh, 2007). Prior to processing, a Gibbs ringing correction (Kellner et al., 2016) was applied to the DW-SSFP, TIR, and TSE datasets. Quantitative T1 and T2 maps were generated from the TIR and TSE datasets assuming monoexponential signal evolution. A B1 map was generated from the AFI data following the methodology described in Yarnykh (2007). All coregistrations within and between imaging modalities were performed with FLIRT (Jenkinson and Smith, 2001; Jenkinson et al., 2002) via a six degrees of freedom (translation and rotation) transformation. The DW-SSFP data, along with the T1, T2, and B1 maps were fitted with the full DW-SSFP signal equation (Buxton, 1993) to both a diffusion tensor model and a ball-and-two-stick model using cuDIMOT (Hernandez-Fernandez et al., 2019).

#### Gibbon, macaque monkey, squirrel monkey, and lemur data

Whole-brain samples were obtained from one gibbon (*Hylobates lar*, male, 5.5 years old), three rhesus macaques (*Macaca mulatta*, between 11 and 15 years old, one female, two males), three squirrel monkeys (*Saimiri boliviensis*, between 2 and 19 years old, one female, two males), and three ring-tailed lemurs (*Lemur catta*, between 3 and 11 years, three males). The samples were obtained from Copenhagen Zoo (gibbon, squirrel monkeys, and lemurs), the London Zoological Society (squirrel monkey), and the University of Oxford’s Biomedical Sciences (macaques). The samples were acquired from deceased animals that died of causes unrelated to this research project. Details of sample preparation, scanning procedure, and data preprocessing have been described previously (Roumazeilles et al., 2022; Bryant et al., 2023). In brief, all brains were extracted and fixed within 24 h after the death of the animal. All brains were rehydrated in a PBS solution 1 week prior to scanning and placed in fomblin or fluorinert for the scanning procedure. The diffusion-weighted MRI data were acquired from the whole brain using a 7 T preclinical MRI scanner (Varian). The scanner bore diameter is 210 mm, the gradient coil references are the following: 205_120_HD (Varian) with a Gmax of 50 G/cm. The radiofrequency coil was made by RAPID Biomedical and is a birdcage transmit receive coil with 72 mm ID. We used a 2D diffusion-weighted spin-echo multislice protocol with single-line readout (DW-SEMS; TR, 10 s; TE, 26 ms; matrix size, 128 × 128 with a sufficient number of slices to cover each brain; resolution for ring-tailed lemurs, 0.5 mm isotropic; resolution for gibbon and macaques, 0.6 mm isotropic; resolution for squirrel monkey, 0.5 mm isotropic). A total of 16 nondiffusion-weighted (*b* = 0 s/mm^2^) and 128 diffusion-weighted (*b* = 4,000 s/mm^2^) volumes were acquired with diffusion encoding directions evenly distributed over the whole sphere (single-shell protocol). Diffusion tensors were fitted to each voxel using DTIFIT (Behrens et al., 2003). Subsequent preprocessing and analytical steps were specific for each species and are described in the following sections. Voxel-wise model fitting of diffusion orientations was performed using FSL’s BedpostX to fit a crossing fiber model to the data (Behrens et al., 2007).

### Probabilistic tractography

Tractography was run using FSL’s probtrackx2 using the following parameters. Probabilistic tractography does not suffer from some of the problems inherent in deterministic methods, in that it allows us to visualize the full distributions of reconstructed pathways. We adjusted some parameters to the size of the brain (Jbabdi et al., 2015), but most importantly we normalized each tract to its maximum within the sample, such that we could obtain a comparable distribution across species. The parameters, similar to our approach in previous studies, were kept as similar as possible across species for consistency and translatability: human, maximum of 3,200 steps per sample, 10,000 samples, step size of 0.25 mm, and curvature threshold of 0.2; macaque, maximum 3,200 steps per sample, 10,000 samples, step size of 0.1 mm, and curvature threshold of 0.2; chimpanzee and gorilla, maximum of 3,200 steps per sample, 10,000 samples, step size of 0.1 mm, and curvature threshold of 0.2; gibbon, maximum of 3,200 steps per sample, 10,000 samples, step size of 0.1 mm, and curvature threshold of 0.2; squirrel monkey, maximum of 3,200 steps per sample, 10,000 samples, step size of 0.1 mm, and curvature threshold of 0.2; and ring-tailed lemur, maximum of 3,200 steps per sample, 10,000 samples, step size of 0.1 mm, and curvature threshold of 0.2. It should be noted that diffusion MRI cannot distinguish between direct and indirect connections, so the identified fibers could represent either type of projection and travel in either direction.

### Comparative probabilistic tractography protocol

Probabilistic tractography of the anterior limb of the AmF, the UF, and the frontal segment of the CB was performed in diffusion space. Descriptions of anatomical locations were made by reference to previously published atlases (Paxinos et al., 2000; Nieuwenhuys et al., 2008), tract-tracing studies (Fudge et al., 2002; Schmahmann and Pandya, 2006; Lehman et al., 2011), and diffusion-tractography studies (Croxson et al., 2005; Jbabdi et al., 2013).

In all species seed (Suppl. Fig. 1), exclusion and waypoint masks were reconstructed in the same way with regard to established white and gray matter regions. The seed mask of the AmF was drawn in the subcommissural white matter perforating the substantia innominata, a region between the dorsal amygdala and the bed nuclei of the stria terminalis (BNST). The mask was located medially to the ventral pallidum (VP) and sublenticular extended amygdala (SLEA) and dorsally to the nucleus basalis of Meynert (NBM; Paxinos et al., 2000; Mai et al., 2015). Its position carefully aimed to reproduce macaque tract-tracing studies (Fudge and Haber, 2001; Schmahmann and Pandya, 2006; deCampo and Fudge, 2013). In accordance with the local trajectory of the AmF (as estimated by tract tracing), the seed mask was constrained to contiguous voxels showing high fractional anisotropy in an anterior–posterior direction. UF was seeded axially in the anterior temporal lobe, in the white matter rostrolateral to the amygdala. An axial seed mask was chosen to account for the strong curve of the fibers from dorsal–ventral to anterior–posterior orientation to the orientation as they enter into the frontal lobe (Folloni et al., 2019a). The CB seed mask was drawn in a coronal plane capturing the white matter dorsal and medial to the corpus callosum, within the cingulate gyrus. To increase the anatomical accuracy of our tracts, two additional tracts, the extreme/external capsule complex and the anterior limb of the internal capsule, were created and their seed masks used as exclusion regions for AmF, UF, and CB. Excluding fibers related to these latter two tracts is important because they are expected to run or terminate in close proximity to AmF, UF, and CB.

To constrain the tractography algorithm, exclusion masks were drawn in all species to exclude false-positive results in areas of high crossing fibers as follows: (1) within the basal ganglia to avoid picking up spurious subcortical tracts; (2) posterior to the seeds to prevent the projections from running backward as the prefrontal cortical streamlines were the focus of this study; (3) an axial slice at the level of the superior temporal gyrus to prevent tracts from running in a ventral direction in an unconstrained manner (except for fibers reaching the amygdala); (4) on the axial–coronal slices cutting across the thalamus, basal ganglia, and corpus callosum to exclude subcortical and callosal projections; (5) in the dorsal cingulate cortex to avoid leakage of tracts to nearby bundles as a result of high curvature of the tracts (except for CB tracking); (6) an L-shaped coronal mask from the paracingulate cortex and the inferior frontal sulcus to the vertex to exclude tracking in the superior longitudinal fascicle; and (7) a mask excluding the opposite hemisphere to only track ipsilateral tracts. In humans, an exclusion mask of the CSF was used in each subject to prevent fibers from jumping across sulci during tracking. Streamlines encountering any of the exclusion masks were excluded from the tractography results. A coronal waypoint section was drawn in the frontal lobe at the level of the caudal genu of the corpus callosum to ensure that the fibers emanating from the seeds were projecting to the PFC.

Streamlines were seeded from each voxel within each subject's seed mask within the body of the tract of interest. We aimed to keep all seeding and tracking parameters constant across tracts and species, only adjusting the step size based on the brain size and white-matter density. Each streamline followed local orientations sampled from the posterior distribution given by BedpostX (Behrens et al., 2007). A visitation map or tractogram was constructed for each individual in order to allow comparison of these maps between tracts, subjects, and species. To counteract underweighting of distant voxels, the tractograms were log-transformed. Subsequently, to allow direct comparison across tracts, subjects, and species, independent of the brain size and scan resolution, we further standardized the data by dividing a tract's voxel values by the 75th percentile value across the tract, thereby removing potential bias of differences in numbers of streamlines. In both human and macaques, the focus of the investigation was on the tracts' connections with PFC in both hemispheres. If multiple subjects per species were available, the normalized tractogram was then warped to each species' group template and averaged into a species-specific tractogram. For the purposes of visualization only, the normalized tractograms were subsequently thresholded. Figures were created with BioRender.com and FSL (Smith et al., 2004).

### Data sharing statement

Data were analyzed using tools from FSL (Smith et al., 2004), which are available online at fsl.fmrib.ox.ac.uk. Human MRI data were obtained from the HCP (Van Essen et al., 2013) and available online from humanconnectome.org. The nonhuman primate MRI data are available from the Digital Brain Bank (Tendler et al., 2022) online at open.win.ox.ac.uk/DigitalBrainBank.

## Results

We reconstructed the principal connections between the limbic system and the PFC in the human brain and compared their organization with that in great (chimpanzee and gorilla) and lesser (gibbon) apes, an Old World (macaque monkey) and a New World monkey (squirrel monkey), and a prosimian (ring-tailed lemur). The evolutionary relationship between these species is shown in Figure 1. The goal was to assess how these connectional systems may have varied or remained consistent among primates. Our anatomical reconstruction focused on the temporal–prefrontal rostral branch of the AmF, the UF, and the anterior section of the CB. We first describe reconstructions of all three tracts in the human and macaque monkey in detail and then use them as baselines for subsequent descriptions of similarities and differences among the other species investigated. Importantly, here we defined anatomical and comparative approaches to reconstruct the anterior connections of the CB, AmF, and UF as they innervate the frontal and temporal cortex. The approach used in each species was designed to be as similar as possible, allowing us to directly compare the organization of these fiber tracts within the methodological limitations. This follows prior studies that describe different brains in terms of directly comparable, homologous features (Elston et al., 2011; Mars et al., 2018; Preuss and Wise, 2022; Bryant et al., 2023).

### Reconstruction of limbic–frontal connections in macaque and human

**Figure 2.**
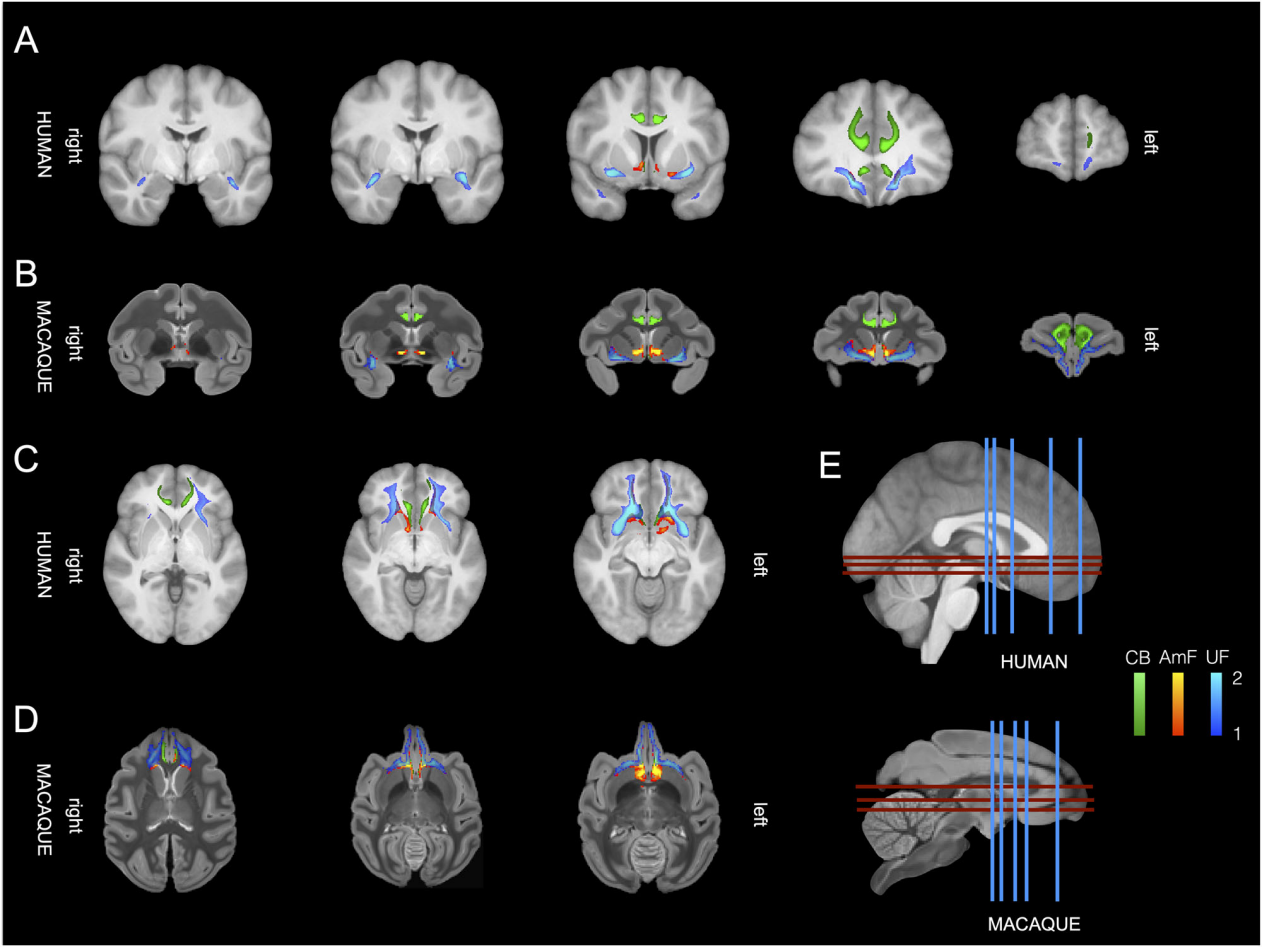
Limbic–frontal connections in macaque monkeys and humans. The anterior limbs of the AmF (red–yellow), UF (blue–light blue), and CB (green) are displayed across the two species on five coronal brain slices (***A,B***) and three axial brain (***C,D***) slices showing their respective interactions and organization in limbic and PFC. Location of slices from ***A–D*** are reported in ***E*** for the human brain (top) and macaque monkey brain (bottom).

The UF is a C-shaped fiber bundle that reciprocally connects the temporopolar cortex and amygdala with the orbital and ventrolateral PFC. In our reconstruction, in a ventral section, the macaque UF extended along the outer surface of the temporal lobe and curved through the superior temporal gyrus (Fig. 2). While the main direction of fibers in this region is from dorsal to ventral, some fibers branched along a medial-to-lateral axis toward the amygdala, thus connecting adjacent fibers near the anterior temporal region and the lateral PFC (Fig. 2). At the level of the limen insulae, UF fibers traveled in the white matter ventral to the claustrum, ventrolateral to the putamen and globus pallidus, and medial to the insular cortex (Fig. 2). The human UF maintained a similar C-shaped structure projecting between the rostromedial temporal regions and the ventrolateral PFC (Fig. 2). Like the macaque temporal UF, it exhibited an anatomical organization primarily along a dorsal–ventral gradient but with lateral fibers extending toward amygdaloid nuclei, parahippocampal areas, and rhinal areas (Fig. 2).

**Figure 3.**
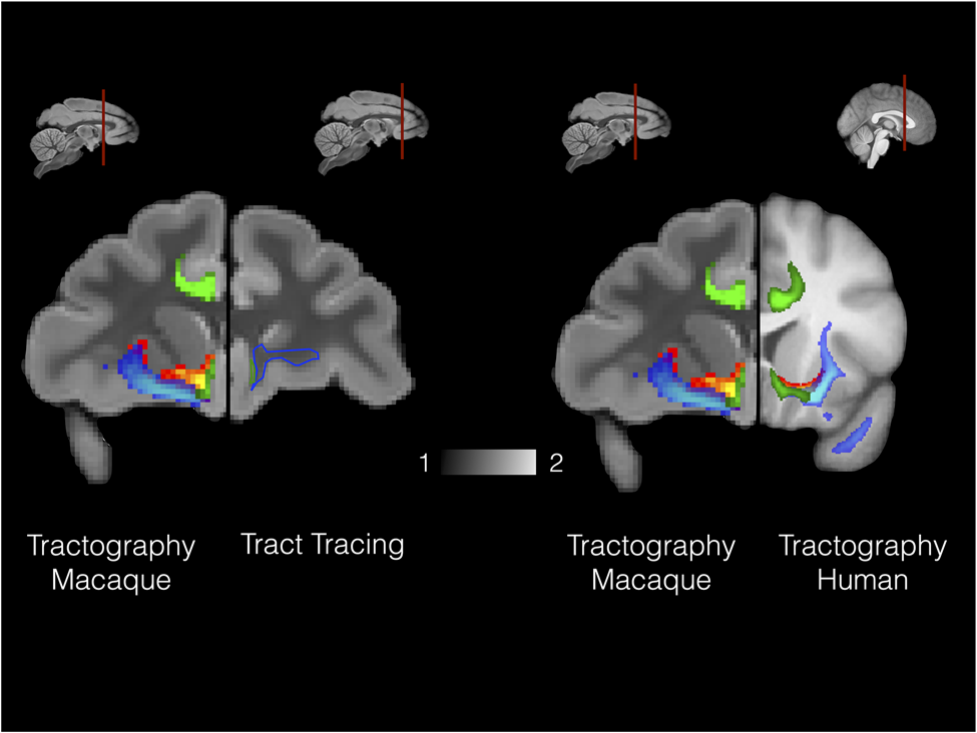
Histology–diffusion MRI comparison of connections. Left part of the figure shows tractography reconstruction (left part) and tract-tracing reconstruction [right part, reconstructed from Heilbronner and Haber (2014) their Figure 15] of amygdala–cingulate–prefrontal connections in the macaque. Right part of the figures shows tractography reconstruction in the macaque monkey (left part) and human (right part). In all brains the AmF (red–yellow), UF (blue–light blue), and CB (green) are shown.

**Figure 4.**
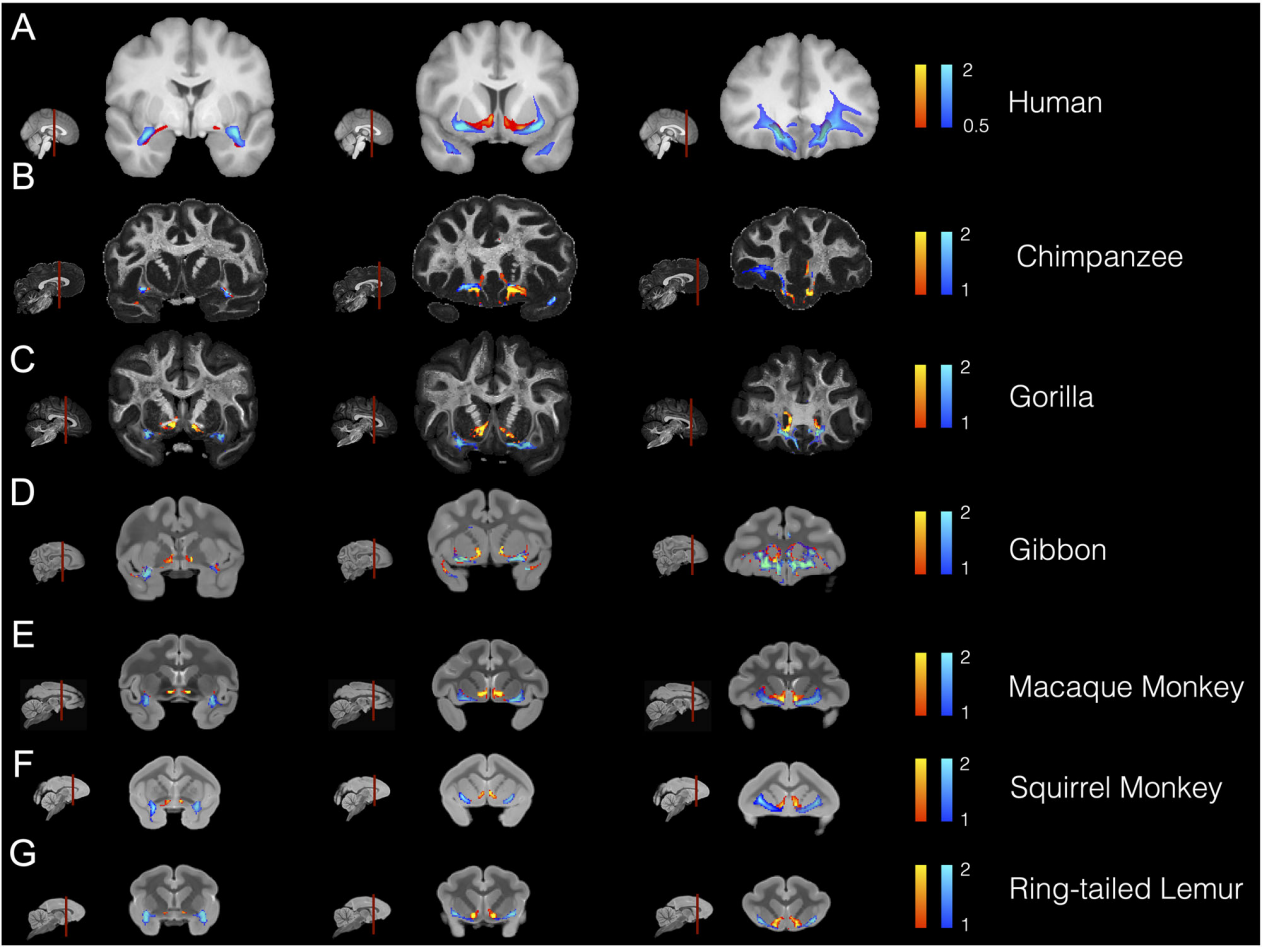
Coronal views of temporal–frontal connections in all species. The anterior limb of the AmF (red–yellow) and the UF (blue–light blue) are displayed across each species on three coronal brain slices showing their respective interactions and organization along a posterior (leftmost column)—anterior (rightmost column) gradient. The brains are displayed from the species most closely related to humans (top row) to the least related (ring-tailed lemur, bottom row).

**Figure 5.**
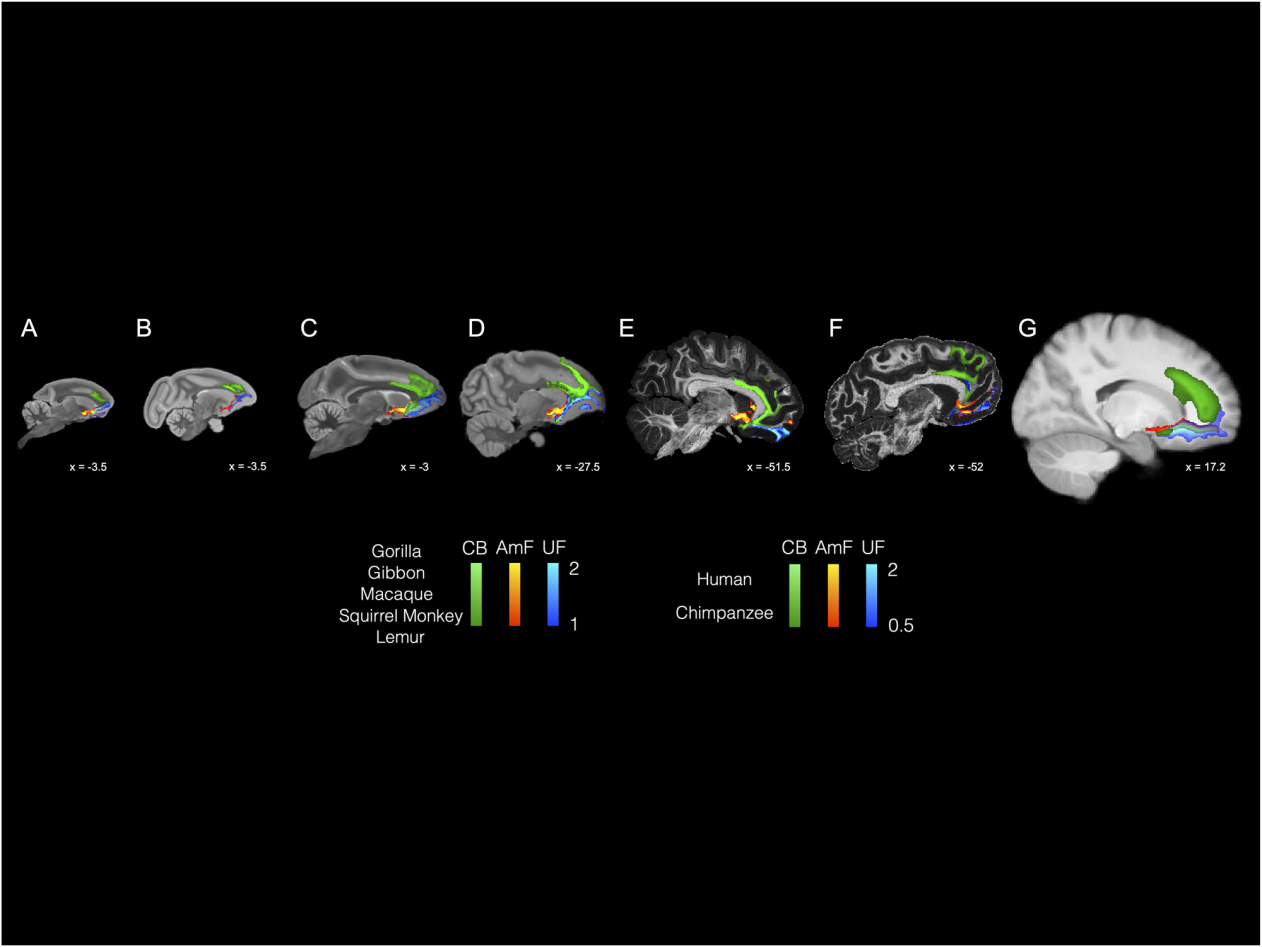
Organization of temporal–cingulate–frontal connections across all species. The anterior limbs of the AmF (red–yellow), UF (blue–light blue), and CB (green) are displayed across each species on a sagittal brain slice showing their organization within cingulate and prefrontal cortices, striatum, and basal forebrain. The brains are displayed in the following order from the left to right: ring-tailed lemur (prosimians), squirrel monkey (New World monkey), macaque monkey (Old World monkey), gibbon (lesser ape), gorilla (great ape), chimpanzee (great ape), and human.

Within the macaque PFC, the UF exhibited strong connectivity with the white matter bordering the caudolateral orbitofrontal cortex (OFC), as well as opercular and inferior frontal areas. Additionally, a subset of fibers extended alongside the subgenual cingulate area and the medial bank of the posterior OFC (Figs. 3–5). In this region, the more medial UF fibers joined with the fibers of the AmF and the CB to innervate the white matter of the anterior cingulate cortex (ACC; Figs. 3–5). Overall, the primary portion of the UF in the ventral PFC spanned across the lateral, central, and medial orbital gyri, forming a complex network within the OFC that extended into the frontopolar cortex (FPC; Figs. 2,4,5). Similarly, in the human, at the junction of the prefrontal and temporal cortices, the UF closely followed the insular cortex and areas within the caudal region of the inferior frontal gyrus (Fig. 2). Along the basal ganglia, the human UF encompasses the lateral ventral striatum and extends to a more lateral section of the ventral PFC (Fig. 2). In this region, the main body of the macaque and human UF continues to traverse laterally, providing extensive innervation to the white matter of the ventrolateral PFC. Furthermore, a subset of fibers also courses more medially, adjacent to the subgenual ACC (sACC), and more prominently toward the posterior OFC alongside the AmF and the CB (Figs. 3–5). Within the anterior region of the ventral PFC, both macaque and human UF extends across medial and lateral orbital areas. A prominent bundle of fibers occupies a substantial portion of the white matter in the lateral OFC and adjacent cortical areas on the ventral surface of the brain in both species. Another branch of the UF extends further rostrally in the white matter adjacent to the FPC in both macaque and human brains (Figs. 2, 4, 5).

In the macaque brain, the frontal portion of the AmF was represented by a thin group of fibers that ran in the white matter adjacent to the longitudinal fissure. At the level of the anterior commissure (AC), the fibers in the medially projecting limb of the AmF pathway traveled within the white matter dorsal to the amygdala, between amygdala nuclei, SLEA area, and medial longitudinal fissure (Figs. 2, 4). In this species, one set of AmF fibers ran in the white-matter region between the NBM, the lentiform nucleus, the VP, and the piriform cortex. These medial AmF fibers divided into different sets of projections. Some extended downward to innervate the anterior hypothalamic areas near the paraventricular–supraoptic nuclei, while others coursed dorsally to connect the amygdala with the thalamus, the BNST, and the septum of the macaque monkey. Another set of macaque AmF fibers ran in the white matter surrounding the substantia innominata, VP, and the lower bank of the AC, entering, further rostrally, into the white matter beneath the ventral striatum/nucleus accumbens (Figs. 2, 4). These AmF pathways were found in both macaque monkeys and humans. Despite the strong mediolateral primary diffusion direction characterizing the AC, some weaker AmF fibers that overlapped with the AC were still detectable in our tractography data within both macaque monkey and human brains. It is important to note that, as the AmF passes through the basal forebrain and beneath the striatum, it is likely to interact with other connectional systems.

Eventually, the anterior fibers of the AmF of macaques and humans extended into the orbital cortex and into the rostral PFC by running in the white matter adjacent to the sACC (or Brodmann Area 25) and the adjacent areas in the subcallosal cortex (Figs. 3–5). Near the sACC, the AmF of macaques and humans split into two subsections. The first subsection ran upward alongside the CB in the anterior cingulate sulcus and the surrounding white matter (Fig. 5). The second branch, on the other hand, stretched between the sACC and the FPC, passing underneath the medial OFC between the gyrus rectus and the medial orbital gyrus (Figs. 2, 4, 5).

Frontal connections of the macaque and human CB project along the full extent of the ACC. This bundle is composed by a mixture of fibers entering the large CB briefly to reach nearby areas or longer-distance connections reaching distal brain regions. Core CB fibers surround the corpus callosum throughout its course in PFC in both species. A subsection of fibers in rostral CB exits the main bundle and contains axons toward and arriving from both cortical and subcortical areas (Fig. 5). At the level of subcallosal and OFC, in both macaques and humans, CB fibers interact with axons traveling in the AmF and UF (Figs. 3, 5). Here, CB fibers either terminate by innervating adjacent areas or continue coursing in a rostral direction toward the anterior PFC and FPC. In both species, another set of connections continues running along the genu of the corpus callosum, next to Areas 23, 24, and 32, and more dorsally, in the CB branches extending into dorsal ACC regions, premotor cortex, lateral PFC (partly through intermixing with UF fibers), and medial–dorsal PFC Areas 8 and 9 (Fig. 5).

Overall, these results, obtained by probabilistic tractography accounting for multiple possible fiber orientations (Behrens et al., 2007), show that AmF, UF, and prefrontal CB fibers exhibit a high degree of similarity between macaque monkeys and humans (Figs. 3, 4). In both species, fibers from the three bundles merge and intertwine at the level of the subgenual cingulate cortex while partially separating and running independently in anterior prefrontal and temporal cortices (Figs. 3–5). The protocols employed here to reconstruct UF, AmF, and CB in macaque and human were based on Folloni et al. (2019a), tailoring them to obtain results best matching those previously observed using macaque tracer data (Heilbronner and Haber, 2014; Oler et al., 2017). This enabled us to avoid false positives and false negatives that may be created when employing tractography algorithms and ensured the truthfulness of the reconstructed bundles. Figure 3 shows the macaque and human tractography results side by side with previously published tracer data to corroborate these original findings. Having established a protocol that allowed both matching of macaque tractography data with established macaque tracer data and matching of macaque and human data, we felt confident using these protocols in chimpanzee, gorilla, gibbon, squirrel monkey, and ring-tailed lemur. We describe the course of the tracts in these species at the levels of the anterior temporal cortex, subcallosal frontal cortex, and more anterior PFC.

### Course of the AmF and UF between the limbic/temporal and frontal cortex across all species

The most prominent shared feature observed across lemur, squirrel and macaque monkey, gibbon, gorilla, chimpanzee, and human brains is their widespread connectivity between prefrontal and anterior temporal areas, both subcortical medial and lateral cortical structures (Figs. 3–5). Consistently across all the species examined, fibers connect the amygdala, piriform, and inferior temporal cortices on the temporal side with medial and lateral regions in anterior PFC on the opposite end. As the anterior limb of the AmF and the UF travel between the temporal and frontal poles, these bundles innervate striatal, insular, basal forebrain, ventromedial, and ventrolateral PFC areas. Consistently across all the species examined, the AmF exits the amygdala via the medial wall of the temporal lobe and then runs dorsally toward limbic, striatal, and prefrontal structures along other forebrain and thalamic fibers. The UF, instead, unfolds as a curve between the anterior temporal lobe and PFC carrying prefrontal, forebrain, amygdalar, rhinal, piriform, parahippocampal, and temporal associative projections.

A clear distinction between AmF and UF is observable across the six primate species at the level of the amygdala, temporal cortex, and pericommissural white matter, showing the dichotomous mediolateral organization in striatal and basal forebrain we see in macaque and human (Fig. 4). The AmF occupies the white matter perforating the VP and expanding underneath the ventral striatum, primarily branching toward the anterior ventromedial striatum, rostrally, and the hypothalamus, bed nuclei, stria terminalis, and septum, medially. UF fibers course through the subcapsular white matter along the ventral insular cortex and putamen. A few fibers leave the main body of the UF and merge with the AmF, approaching from a central position. This ventral–dorsal intertwining of the two paths is observable across all primate brains examined and becomes complete when AmF and UF approach the sACC. In the white matter innervating the striatal and basal forebrain territory, however, AmF and UF in the gibbon overlap to a higher degree than in the other species, where they were still clearly distinguishable. Interestingly, the mediolateral organization of UF and AmF is also preserved in the strepsirrhine lemur despite the anatomical expansion of the temporal lobe and of the PFC in anthropoids (Gurche, 1982; Passingham and Wise, 2012; Ni et al., 2019; Eldridge et al., 2021).

### Level of the subcallosal cortex

**Figure 6.**
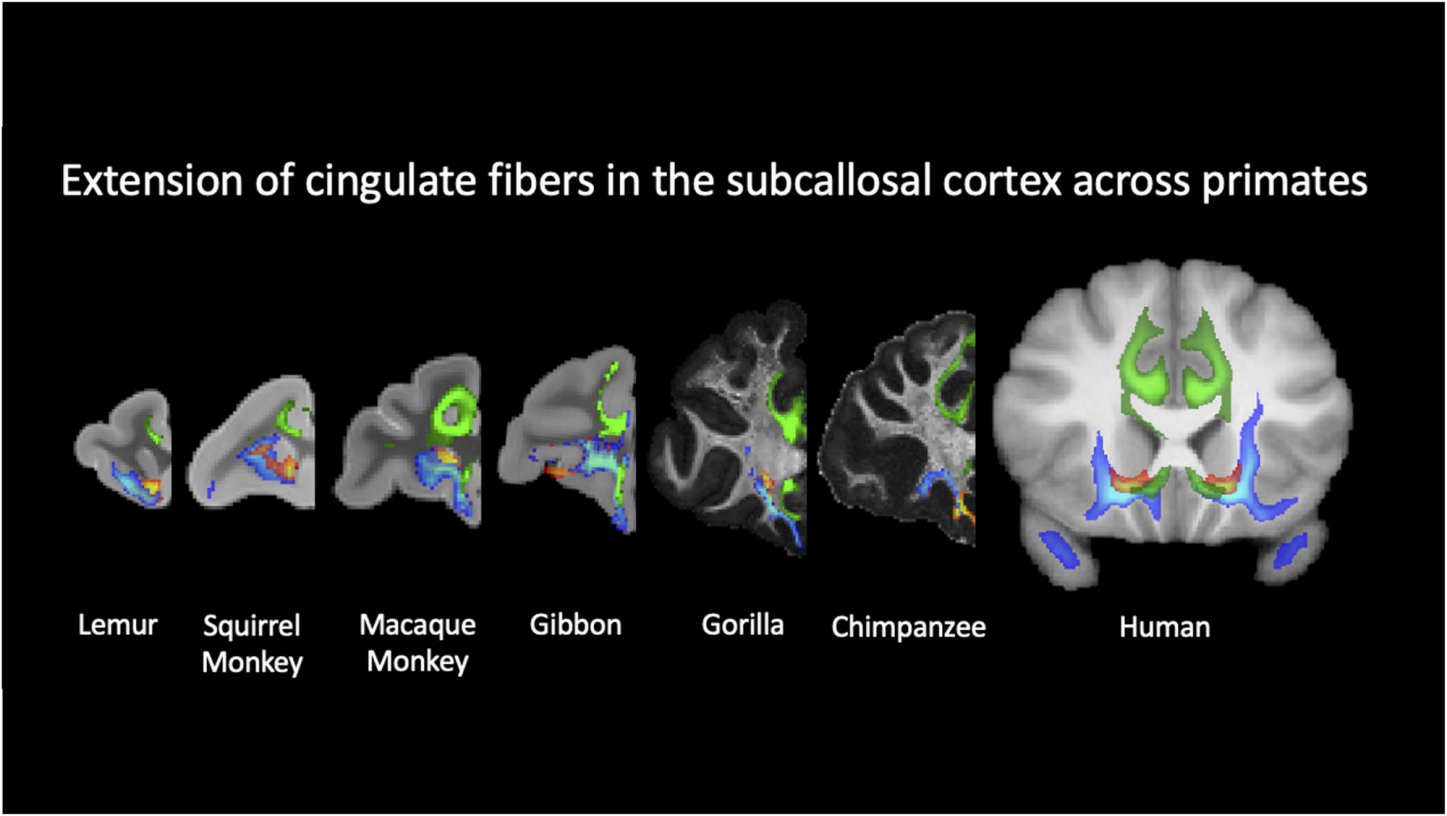
Evolutionary expansion of cingulate projections into the subcallosal cortex across primates. Extension of CB (green), AmF (red–yellow) and UF (blue) into the subcallosal part of the ACC from prosimians to humans. Shown from the left to right are representative coronal slices from the lemur, squirrel monkey, macaque monkey, gibbon, gorilla, chimpanzee, and human, aligned to emphasize homologous subcallosal landmarks. Cingulum fibers can be observed in all primates from the macaque monkey to the human brains but not in lemurs and New World squirrel monkeys. Organization and extension of amygdalofugal and UF are preserved across all species.

Fibers coursing alongside the sACC, underneath the ventral floor of the genus of the corpus callosum, display a common organization across Old World monkey, lesser and great ape, and human brains. After coursing through the temporal lobe and near the ventral striatum, AmF and UF approach the subgenual cortex from a medial and lateral position, respectively. This is also where the main body of the CB joins the territory of AmF and UF, but its main body runs primarily medial to AmF and UF (Figs. 3, 5, 6). In both the macaque and human brains, the CB fibers merge with fibers from AF and UF in this region, confirming results from previous tract-tracing studies (Haber and Behrens, 2014).

In caudal sACC, AmF and UF not only begin sharing important portions of white matter with CB, but they also start merging into a single limbic bundle innervating the ventral PFC and anterior temporal cortex, in addition to the medial cortex at their extremes. Although AmF and UF here begin merging into a single connectional system as they interacted with CB, they maintained a mediolateral organization until they approach posterior OFC. The most dorsomedial WM territory surrounding the sACC is primarily occupied by fibers carried by an additional dorsal limbic tract, the CB. Within the PFC, this tract carries fibers running in the middle, anterior, and subgenual cingulate cortex (Figs. 5 and 6).

**Figure 7.**
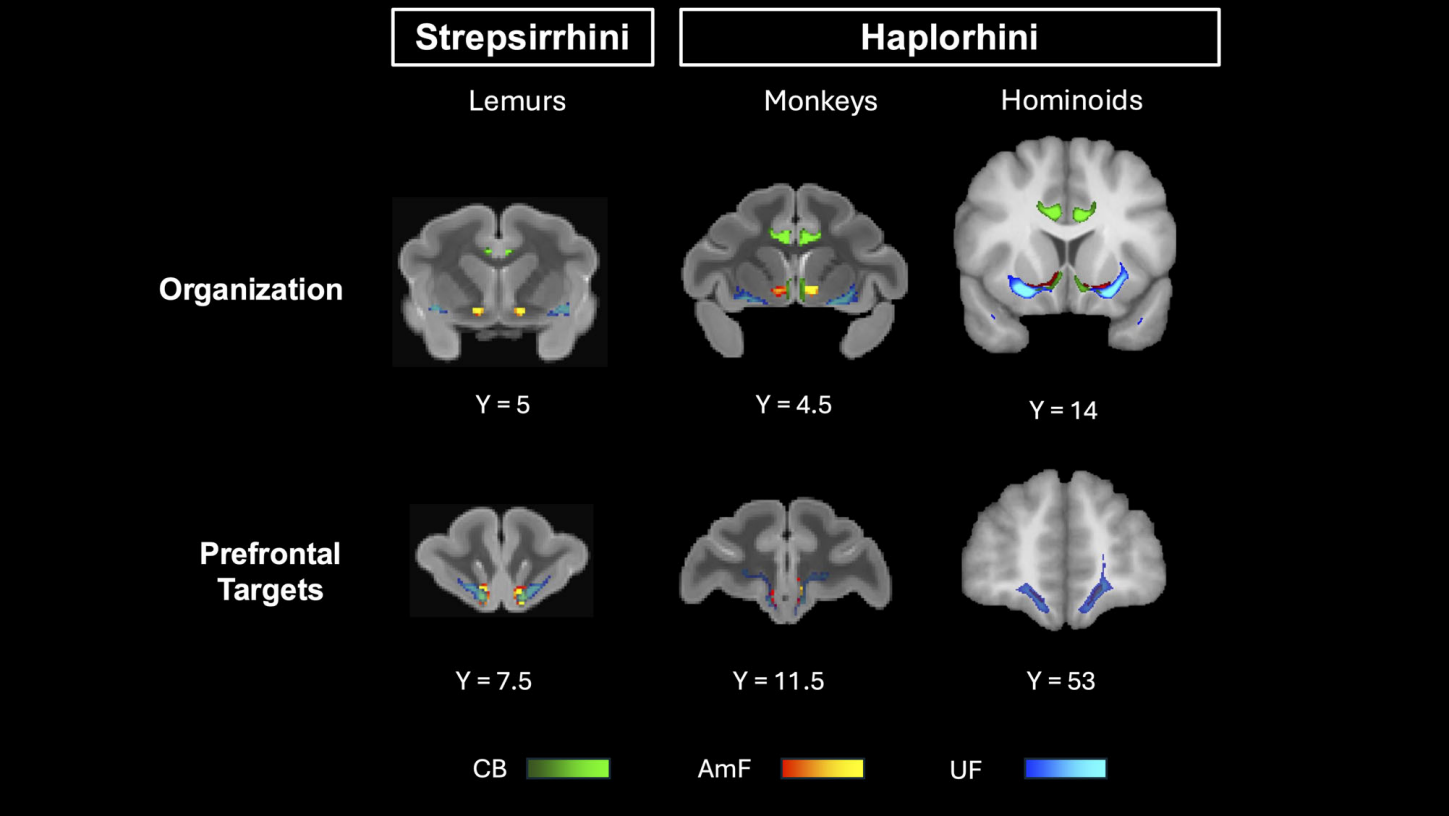
Principles of organization and prefrontal targets across strepsirrhini and haplorhini. Organization and prefrontal targets of CB (green), AmF (red–yellow), and UF (blue) across strepsirrhini (lemurs, left) and haplorrhine (macaque monkeys and humans, right). AmF and UF organization is similar across the two suborders or primates, with separation in more caudal regions and convergence in the anterior frontal cortex. Extension of the CB fibers in the subcallosal cortex, however, differs between the two suborders.

Interestingly, CB fibers did not extend into the caudal sACC as clearly as in the prosimian (ring-tailed lemur) and New World monkey (squirrel monkey), but we could still observe a similar organization of AmF and UF as described above for the macaque monkey, gibbon, gorilla, chimpanzee, and human brains (Figs. 6, 7). This pattern was consistent even when adjusting for different values of the curvature parameter used in our analyses aiming to account for the anatomy of the genu of the corpus callosum in these two species.

### Level of anterior PFC

Thus far, the organization of UF, AmF, and CB is similar across all of the primates studied here. However, PFC has differentially expanded in different primate lineages, showing a relative increase in the size in anthropoid primates (Passingham and Wise, 2012) and perhaps again in the ape and especially the human lineage (Passingham and Smaers, 2014; Donahue et al., 2018). It is therefore of particular interest to examine how these tracts project to anterior PFC.

A merging of the amygdala, temporal cingulate, and prefrontal connections in posterior OFC was clearly observable along all of the representatives of the primate order examined (Fig. 4). As previously reported for macaque and human brains (Folloni et al., 2019a), AmF and UF transition from a dichotomous organization in temporal and subcortical regions to an intertwined bundle in more anterior PFC white matter. Although in medial portions of anterior PFC, AmF and UF occupy a similar portion of white matter, we observed that AmF occupies the territory adjacent to the frontal medial wall, whereas UF extends to lateral ventral areas.

In anterior PFC, AmF and UF fibers coursed together along the medial orbital and frontopolar cortices consistently across lemurs, squirrel and macaque monkeys, gibbons, gorilla, chimpanzees, and humans regardless of their frontal lobe expansion (Fig. 4). In addition, UF fibers branched out to caudolateral OFC, ventrolateral PFC, as well as opercular and inferior frontal areas (Fig. 4). Axons from both AmF and UF reached granular PFC in the primate species that possess this cell type. Anatomical interactions among AmF, UF, and CB fibers in caudal–ventral PFC were shared across all species, except in the gibbon, where AmF and UF converged more posteriorly, beginning in the transition territory between the subgenual and orbital cortex. Although we cannot entirely rule out the influence of false positives given the small sample size for this species, it is noticeable that the gibbon is the only species among those examined here that primarily lives in very small groups and spends the least amount of time in socially and emotionally bonding behaviors (Cunningham and Mootnick, 2009; Guan et al., 2018). This peculiarity in social behavior, either it being environmentally induced or inherent in the gibbon nature, may be associated with our finding that there is a difference in AmF and UF organization in posterior OFC for this species.

## Discussion

**Figure 8.**
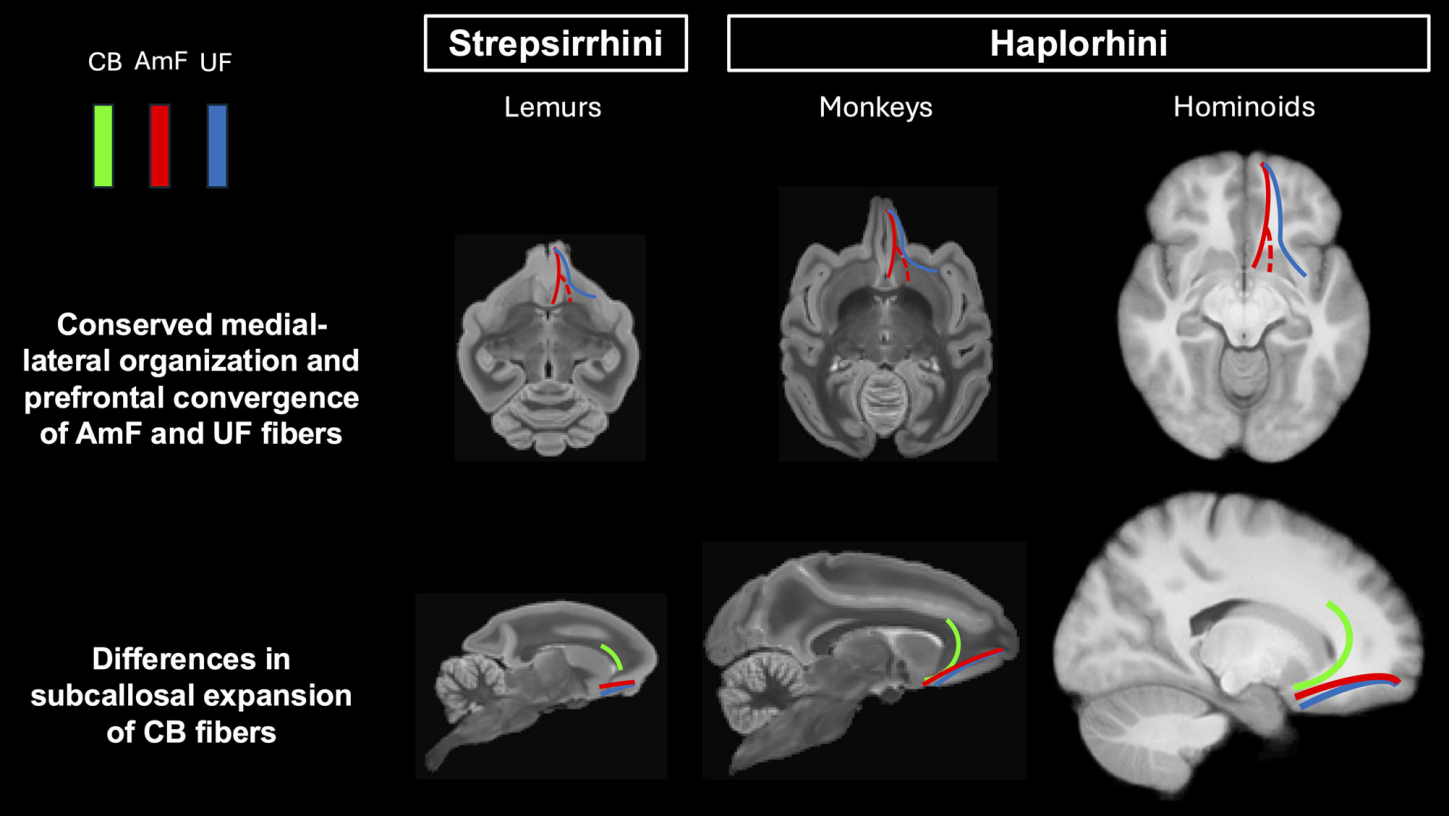
Schematic diagram of similarities and differences across strepsirrhini and haplorhini in the limbic–prefrontal connectome. Schematic template representing the organization of AmF (red), UF (blue), and CB (green) in the ventral PFC in the lemur (left), monkey (middle), and human brains displayed on an axial (top row) and sagittal slice (bottom row). The dashed line (red) shows the subcortical AmF fibers running underneath and through the ventral striatum.

In this study, we reconstructed the limbic–prefrontal connections in species from all major branches of the primate radiation. By using the same methodology, we showed that the overall organization of AmF, UF, and CB is evolutionarily conserved but with some species-specific variances (Fig. 8). This is important given the need to improve translation from animal models to humans for developing new brain treatments. Notably, our diffusion-tractography results reveal that the anatomy of limbic–frontal fibers resembled previous tracer investigations focused on these specific tracts in macaque monkeys (Kunishio and Haber, 1994; Fudge and Haber, 2001; Fudge et al., 2002; Lehman et al., 2011; Heilbronner and Haber, 2014; Oler et al., 2017) and diffusion imaging studies that compared only a subset of the species investigated here (Thiebaut de Schotten et al., 2012; Folloni et al., 2019a; Roumazeilles et al., 2020).

Primate brains, including humans, share a general organizational plan but exhibit species-specific adaptations (Preuss and Kaas, 2003). Temporal and frontal cortices expanded in hominoids (Chaplin et al., 2013; Donahue et al., 2018; Braunsdorf et al., 2021), accompanied by increased organizational complexity and differentiation in both gray and white matter (Eichert et al., 2019; Barrett et al., 2020; Roumazeilles et al., 2020). To our knowledge, this is the first study to compare the organization of specific white-matter tracts across all major branches of the primate family tree. Previous work has either examined more global connectivity measures (Ardesch et al., 2022; Suarez et al., 2022) or focused on a limited number of species (Barrett et al., 2020; Roumazeilles et al., 2020).

Despite anatomical heterogeneities in temporal and frontal cortex across primates, the overall organization of the AmF, UF, and CB appears conserved. In all species, amygdala–temporal–frontal connections travel in three distinct tracts with similar courses and relative topology. This suggests that their organization represents a shared anatomical feature, possibly originating in early primates. Interspecies similarities in connectivity within the anterior temporal and frontal lobes may be indicative of how the environment shaped white-matter organization in early primates. Furthermore, these shared features may also relate to common foraging strategies across primates, which rely heavily on visual foraging and associative learning (Eacott and Gaffan, 1992; Gutnikov et al., 1997; Vogt, 2005; Murray and Wise, 2010; Passingham and Wise, 2012; Chau et al., 2015; Joyce and Barbas, 2018; Fouragnan et al., 2019; Wittmann et al., 2020; Bongioanni et al., 2021; Folloni et al., 2021).

Reconstructing fiber bundles in the inhomogeneous tissue such as cingulate, striatal, and ventral prefrontal regions is challenging because fractional anisotropy does not fully capture tract integrity. This can produce false negatives or positives in deterministic tractography. In contrast, the probabilistic tractography approach used here, informed by prior anatomical knowledge, reveals a distribution and reconstruction of streamlines consistent with previous tracer studies.

It is important to properly contextualize this result. We show that the organization of two dissociable limbic–prefrontal pathways, the AmF and UF, in the basal forebrain and subgenual cortex, is conserved across primates. This is a particularly intriguing finding in lemurs and squirrel monkeys, which have markedly lower white matter density in this region. Our methodology focused on reconstructing the main bodies of fiber bundles throughout the brain, a reliable approach for cross-species comparisons because it identifies homologous white-matter structures (Mars et al., 2018) and avoids the false positives common when inferring connectivity directly from gray matter (Maier-Hein et al., 2017). This approach underpins tools used to compare white matter architecture across species, development, and individuals (Warrington et al., 2020, 2022). However, this does mean we do not make claims on the precise gray matter areas innervated by these bundles. Indeed, the conserved fiber systems identified in this work exist within neural systems that do show phylogenetic diversity, as mentioned above. A particularly prominent difference is the addition of granular areas in the PFC (Preuss and Wise, 2022). Future work will determine how these converged pathways relate to diverse gray matter architecture (Eldridge et al., 2021) and the implications of this for function (Bramson et al., 2020).

Despite overall conservation, our results suggest a potential difference in innervation of the subgenual cortex across species. Across primate evolution, cingulate fibers increasingly expand within the subcallosal cortex, transitioning from focal inputs in prosimians and New World monkeys to more expanded projections in macaques, gibbons, great apes, and humans. This expansion is accompanied by a widening separation between limbic and frontotemporal pathways ventrally, reflecting increased differentiation of amygdala–cingulate–prefrontal circuits.

Although still poorly understood, the subcallosal cortex is a key hub for mood and affect, as dysregulation of this region is a core feature of patients with mood disorders and anhedonia (Drevets et al., 1997, 2008). Lesions of this area cause deficits in anticipatory arousal (Rudebeck et al., 2014), whereas hyperactivation is associated with autonomic changes and deficits in reward processing (Alexander et al., 2019) and aversive behavior (Alexander et al., 2019, 2020). These findings implicate the subgenual cortex as critical to the orchestration of neural circuits regulating affective behavior and survival (Wood et al., 2023). In most species, the CB interacts in the subgenual cortex with AmF and UF before the three bundles separate into their own specific trajectories. These results replicate tracer studies in Old World monkeys (Vogt, 2005; Heilbronner and Haber, 2014; Joyce and Barbas, 2018). We did not observe the same organization in lemurs and squirrel monkeys: at comparable thresholds, none of the reconstructed cingulum fibers reached the subcallosal white matter, regardless of the curvature parameters used.

It is important to interpret this result carefully. It is unlikely that the cingulum entirely fails to reach caudal subgenual and ventromedial PFC in these species, as tracer work in squirrel monkeys shows fibers in this white-matter region (Leichnetz and Astruc, 1976). However, the density of fibers reaching this territory, as indicated indirectly by the likelihood of the probabilistic tractography reaching there, is much lower in these species. This is important, as the density of connections is a very important predictor of information exchange between areas (Honey et al., 2010). Our results suggest that the growing use of New World monkeys such as the squirrel monkey in translational research (Royo et al., 2021) should be paired with extensive validation studies to confirm that results from other model species replicate.

The overall preservation of the CB, AmF, and UF across prosimians, New and Old World monkeys, gibbons, and great apes underscores a powerful translational opportunity not only in evolutionary terms but also for developing and improving either novel or existing technologies modulating activity in the subcallosal region. This also cautions against directly translating findings between New and Old World monkey models without accounting for species differences. Nevertheless, even in prosimians and New World monkeys, where subcallosal white-matter fibers are reduced relative to macaques and anthropoids, the organization and separation of limbic–prefrontal tracts are still evident. This is notable given the broader divergences in cortical cytoarchitecture, association–cortex expansion, and prefrontal lamination across these lineages. The presence of homologous pathway scaffolds in these evolutionarily distant species indicates that the core architecture supporting affective and cognitive integration is deeply conserved.

The white-matter similarities and differences identified here have direct relevance for therapeutic interventions targeting the subcallosal cortex, including deep-brain stimulation, transcranial ultrasound neuromodulation, and pharmacological approaches. These findings can inform species-specific parameter selection, interpretation of outcomes, and behavioral phenotypes across diverse models. Notably, the reduced extent of cingulate fibers in prosimians and New World monkeys highlights that translational studies in these species must consider not only the pathway presence but also quantitative features such as stimulation trajectory, strength, and depth that may cause similar manipulations to engage amygdala–cingulate–prefrontal circuits differently across species.

This manuscript includes, to our knowledge, the largest set of species in which a specific group of tracts has been reconstructed in detail. Such reconstructions enable quantitative, whole-brain comparisons, now feasible with datasets like the one presented here (Assimopoulos et al., 2025). Although we outline organizational principles across several primate families, future work could broaden species coverage. For example, we included the prosimian *L. catta* but not smaller brained species such as the prosimian galago or the platyrrhine tamarin. Tarsiers, the only nonanthropoid haplorhines, are also interesting, though MRI data are lacking. Many limbic targets appear conserved across mammals (Murray et al., 2011), raising the question of how widely these principles extend. While tract reconstructions in nonprimates remain limited [but see Inglis et al. (2024) and Wright et al. (2018)], the recent release of carnivore MRI data (Boch et al., 2026) provides an opportunity to examine these evolutionary patterns more broadly.

In conclusion, we show that despite differences in phylogeny, life history, behavior, and ecology, the topology of limbic–frontal connections is shared not only across humans, apes, and monkeys but also with simians and prosimians. Although these limbic–frontal connections innervate systems that vary in size and morphology, their overall architecture and course are conserved. The cingulum connections to the subgenual cortex, a key target for brain therapies, are an exception. Together, this work shows how a large-scale comparative approach can help guide translational neuroscience as well as provide insights into brain evolution.
